# COVID-19 Diagnosis on Chest Radiographs with Enhanced Deep Neural Networks

**DOI:** 10.3390/diagnostics12081828

**Published:** 2022-07-29

**Authors:** Chin Poo Lee, Kian Ming Lim

**Affiliations:** Faculty of Information Science and Technology, Multimedia University, Melaka 75450, Malaysia; kmlim@mmu.edu.my

**Keywords:** COVID-19, deep neural networks, chest X-ray, chest radiograph, DenseNet, fine-tuning, pre-trained, CNN

## Abstract

The COVID-19 pandemic has caused a devastating impact on the social activity, economy and politics worldwide. Techniques to diagnose COVID-19 cases by examining anomalies in chest X-ray images are urgently needed. Inspired by the success of deep learning in various tasks, this paper evaluates the performance of four deep neural networks in detecting COVID-19 patients from their chest radiographs. The deep neural networks studied include VGG16, MobileNet, ResNet50 and DenseNet201. Preliminary experiments show that all deep neural networks perform promisingly, while DenseNet201 outshines other models. Nevertheless, the sensitivity rates of the models are below expectations, which can be attributed to several factors: limited publicly available COVID-19 images, imbalanced sample size for the COVID-19 class and non-COVID-19 class, overfitting or underfitting of the deep neural networks and that the feature extraction of pre-trained models does not adapt well to the COVID-19 detection task. To address these factors, several enhancements are proposed, including data augmentation, adjusted class weights, early stopping and fine-tuning, to improve the performance. Empirical results on DenseNet201 with these enhancements demonstrate outstanding performance with an accuracy of 0.999%, precision of 0.9899%, sensitivity of 0.98%, specificity of 0.9997% and F1-score of 0.9849% on the COVID-Xray-5k dataset.

## 1. Introduction

Since 2019, the SARS-CoV-2 virus has caused severe outbreaks of COVID-19 disease to the whole world. The virus is highly infectious; therefore, early detection of COVID-19 symptoms is of real significance to enable immediate isolation and prevention of the virus spreading to the healthy population. Due to the scarcity of publicly available chest radiographs (also known as chest X-rays) of COVID-19 patients, studies on COVID-19 detection from chest radiographs are still limited. Recently, a COVID-Xray-5k dataset with 5000 chest radiographs was compiled by Minaee et al. (2020) [1].

**Figure 1:**
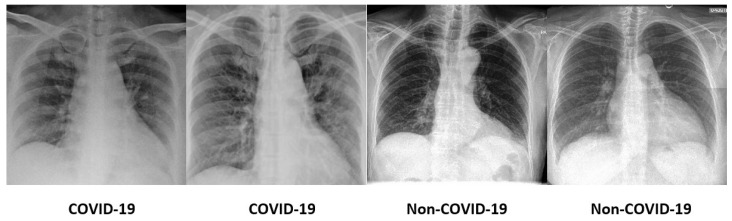
Some sample COVID-19 and non-COVID-19 images from the COVID-Xray-5k dataset.

The dataset is challenging as it is highly imbalanced with 184 COVID-19 images and 5000 non-COVID-19 images. Not only that, the signs in the lungs that indicate the infection by the SARS-CoV-2 virus are hardly perceivable and diversified. Some sample COVID-19 and non-COVID-19 images are shown in Figure 1. In light of this, conventional machine-learning methods that require manual feature engineering to describe the images are ineffective. Therefore, the deep neural networks that are able to autonomously learn the prominent features and perform recognition are preferred in this COVID-19 diagnosis task.

In this work, four deep neural networks are examined, including VGG16, MobileNetV2, ResNet50 and DenseNet201, for COVID-19 diagnosis. The performance of these models was evaluated on the COVID-Xray-5k dataset [1] for COVID-19 diagnosis. Since the COVID-19 chest radiographs are limited, the research faces the challenges of data scarcity and imbalanced class distribution.

Not only that, the mechanism to decide when to stop the training of deep neural networks is essential as it is a determining factor for the generalization capability of the model. Too many training epochs makes the model overfit the training data, while too few epochs lead to underfitting. Although the deep neural networks were pre-trained on the ImageNet, re-training the model on the COVID-19 chest radiograph dataset enables the model to better adapt to the downstream task, particularly COVID-19 diagnosis. In view of this, the following contributions are proposed to address the challenges:

- Due to the limited number of COVID-19 chest radiographs, data augmentation is used to synthesize more samples for model learning. Different variations of COVID-19 chest radiographs, such as random cropping and intensity normalization, increase the number of samples of the COVID-19 chest radiographs. The random cropping and intensity normalization are appropriate as they are intuitively analogous to the different settings in radiography examinations.
- As the sample distributions of COVID-19 and non-COVID-19 classes are highly imbalanced, the class weights are adjusted to be inversely proportional to the sample size in each class. The adjusted class weights reduce the bias and place more emphasis on the minority class, thus, mitigating the imbalanced class distribution issues.
- To circumvent the overfitting and underfitting problems of the deep neural networks, the early stopping mechanism is implemented to terminate the training of deep neural networks after the validation accuracy stops improving for a certain number of epochs. The weights with the highest validation accuracy are then restored and used for testing.
- Training the entire deep neural networks requires large datasets, while training only the final classification layer has the risk of inferior feature representation specific to the downstream task. In light of this, the fine-tuning is performed on the last few layers of deep neural networks to better represent the visual cues of chest radiographs. This is a trade-off between end-to-end training and training only the final classification layer.

## 2. Related Works

This section reviews some existing works that deploy deep-learning models on chest radiographs for COVID-19 diagnosis [2,3,4,5].

Narin et al. (2020) [6] evaluated five deep neural networks—namely, InceptionV3, ResNet50, ResNet101, ResNet152 and Inception-ResNetV2—for the diagnosis of COVID-19 cases using chest radiographs. The authors collected 341 COVID-19 and 2800 healthy chest radiographs from different sources and conducted five-fold cross validation. Among the deep neural networks, ResNet50 achieved the highest accuracy of 96.1%.

Basu et al. (2020) [7] performed domain extension transfer learning on three deep neural networks, which were AlexNet, VGG16 and ResNet50, for COVID-19 diagnosis. The deep neural networks were first trained on a dataset with 108,948 images from the normal and diseased classes to let the model learn to classify two classes. The trained deep neural networks were then fine-tuned on the second dataset that comprised 305 COVID-19, 322 pneumonia, 350 normal and 50 other disease images. The VGG16 model recorded the best performance on the second dataset with 90.13%.

In Jain et al. (2021) [8], three deep neural networks, including Inception V3, Xception and ResNeXt, were evaluated for COVID-19 detection. The dataset was collected from Kaggle with 576 COVID-19, 1583 normal and 4273 pneumonia images. The activation function in the deep neural networks was changed from ReLU to LeakyReLU to avoid inactive neurons. The experimental results suggested that the Xception model obtained the highest F1-score of 0.97.

Five deep neural networks—namely, VGG16, VGG19, ResNet18, ResNet50 and ResNet101 —were adopted in Ismael et al. (2021) [9]. The deep neural networks were utilized as the deep feature extractor. Subsequently, the deep features were fed into the Support Vector Machines (SVM) for classification. The experiments included 180 COVID-19 and 200 normal chest X-ray images. Among the deep neural networks, the combination of ResNet50 and SVM produced the highest accuracy of 94.7%.

Wang et al. (2020) [10] introduced a lightweight COVID-Net with projection-expansion-projection-extension (PEPX) architecture for COVID-19 diagnosis. The network utilized convolution operations of different kernel sizes to extract the features from the chest radiograph images, project the input features into a lower dimension, followed by expansion into a higher dimension, the learning of spatial properties, projecting the features back to a lower dimension and lastly extending the features channel-wise into a higher dimension as the final features. The COVID-Net recorded 93.3% accuracy on the COVIDx dataset with 13,975 chest X-ray images, out of which 358 images belong to COVID-19 class.

An attention-based VGG16 was proposed in Sitaula and Hossain (2020) [11]. The VGG16 was adopted as the backbone with the integration of an attention module in the fourth pooling layer. In the attention module, both the max pooling and average pooling outputs were concatenated to encode the spatial relationship between the regions of interest. The model achieved accuracy of 79.58% on dataset 1 with 1125 images, 85.43% on dataset 2 with 1638 images and 87.49% on dataset 3 with 2138 images.

VGG16 was similarly employed in Ahsan et al. (2021) [12] for COVID-19 diagnosis. The chest X-ray images were filtered by a modified anisotropic diffusion technique to preserve the edges and clean the noise in the images. The cleaned images were then represented by histograms of oriented gradients and CNN. The extracted features were then fused and passed into the VGG16 for classification. The experiments on the dataset consisted of 1979 COVID-19 and 3111 normal images and recorded an accuracy of 99.49%.

Another modified pre-trained Convolutional Neural Network (CNN) was presented in Abbas et al. (2021) [13]. A class decomposition layer that consists of a Decompose, Transfer and Compose (DeTraC) model was added to the pre-trained CNN. Specifically, the class decomposition layer used *k*-means clustering to partition each class in the dataset into several subclasses with new labels. Every subclass was then treated as an independent class, and the predicted labels of these subclasses were fused to produce the final predictions. An accuracy of 93.1% was achieved on a self-collected dataset with 80 normal, 105 COVID-19 and 15 SARS images.

A novel deep neural network known as Corona-Nidaan was proposed in Chakraborty et al. (2021) [14]. The Corona-Nidaan was built of 91 layers with depth-wise separable convolutional layers, batch normalization layers, a global average pooling layer, residual connection as the uniqueness. A two-phase oversampling method was leveraged to produce more samples for the minority classes, thus, addressing the imbalanced class problem. The model achieved 95% accuracy on the self-collected dataset with 245 COVID-19, 5551 pneumonia and 8066 normal images.

Sharifrazi et al. (2021) [15] combined a sobel filter, CNN and SVM for COVID-19 diagnosis. The chest X-ray images were subjected to the sobel filter to extract the edges in the image. The resulting images were then fed into CNN for feature extraction, followed by SVM for classification. Experiments on the self-collected dataset comprising 77 COVID-19 images and 256 normal images yielded an accuracy of 99.02%.

Aslan et al. (2022) [16] extracted the features of chest X-ray images using pre-trained models—namely, AlexNet, ResNet18, ResNet50, Inceptionv3, Densenet201, Inceptionresnetv2, MobileNetv2 and GoogleNet. The extracted features were then passed into k-Nearest Neighbors, Decision Tree, SVM and Naive Bayes for classification. The hyperparameter tuning of the classifiers was done by Bayesian optimization. The experiments were conducted on a self-collected dataset that contains 219 COVID-19, 1341 normal and 1345 viral pneumonia images. The Densenet201 with SVM obtained the highest accuracy of 96.29% on the dataset.

Tangudu et al. (2022) [17] utilized pre-trained MobileNet enhanced with the residual separable convolution (RSC) block. The separable convolution layers in the RSC block were equipped with residual connections to reiterate the gradients flow. The experiments were conducted on the COVID-Xray-5k dataset and recorded an accuracy of 99.71%.

The COVID-Xray-5k dataset was also utilized in Rehman et al. (2022) [18]. The chest X-ray images were first enhanced by the cloud balance contrast enhancement technique. The pre-processed images were then represented as textural and shape features and went through feature selection based on gain ration. The selected features were passed into a bootstrap aggregated extreme learning machine (BA-ELM) for final classification. The experiments on the subset of COVID-Xray-5k dataset with 184 COVID-19 and 3000 healthy images achieved 95.7% accuracy. Table 1 summarizes the existing works with their method, dataset and performance.

## 3. Pre-Trained Convolutional Neural Networks

Generally, the deep neural networks require extremely huge training datasets for the model learning so that the model can perform classification or prediction tasks acceptably. As the training on huge dataset involves high computational resources, researchers turn to adopting pre-trained deep neural networks. For image classification tasks, the pre-trained CNNs are mostly used. Pre-trained CNNs are the CNNs that have been trained on a large image dataset with annotations, such as ImageNet.

The model architecture and weights are then saved to be used for other downstream tasks. The downstream tasks first load the architecture and weights of the pre-trained CNNs. As the downstream tasks usually have different target classes than the dataset that the model was trained on, the final classification layer of the pre-trained model will then be replaced and re-trained with the new target classes of the downstream tasks.

In this study, four pre-trained CNNs are explored for COVID-19 detection using chest radiographs. The pre-trained CNNs include VGG16 [19], MobileNetV2 [20], ResNet50V2 [21] and DenseNet201 [22]. To adapt to the COVID-19 diagnosis task, the final classification layer of the pre-trained model is substituted with a new classification layer and re-trained on the classes in COVID-Xray-5k dataset, i.e., COVID-19 and non-COVID-19. A quick overview of each deep neural network is given below:

- VGG16 comprises multiple blocks of convolutional and max pooling layers. The VGG16 replaces the large size kernels in convolutional layers with smaller 3×3 kernels one after another. The max pooling layer uses 2×2 kernel size.
- MobileNetV2 is an enhancement on MobileNetV1 with a depthwise convolution layer that applies a single convolutional kernel for each input channel. The depthwise convolution dramatically reduces the complexity and computation cost of convolutional neural networks.
- ResNet152 introduced the concept of residual connections where the original input is added to the output of the convolution block as the shortcut path. Adding this connection for gradient flow mitigates the problem of vanishing gradient. ResNet50 is a smaller version of ResNet152 that is frequently used as the starting point for transfer learning.
- DenseNet201 comprises multiple dense blocks. Each layer in the dense block concatenates the inputs from the preceding layers with its own feature maps and passes on to the subsequent layers. This characteristic gives the features richer patterns and varying complexity levels.

**Figure 2:**
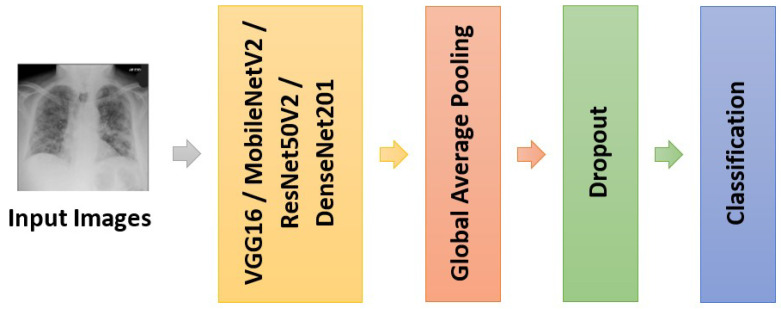
The architecture of the deep neural network for COVID-19 diagnosis.

Prior to the new final classification layer, a global average pooling layer and a dropout layer are added after the pre-trained model. The global average pooling layer flattens the features from the preceding layers and produces a feature map for each class. As deep neural networks are prone to overfitting, the dropout layer added for regularization purposes to improve the generalization capability. The overall architecture of the deep neural network is depicted in Figure 2.

The performance of these pre-trained models is evaluated on COVID-Xray-5k dataset. The COVID-Xray-5k dataset is divided into 2084 training and 3100 test images. Out of 2084 training samples, 84 are COVID-19 images, and 2000 are non-COVID-19 images. On the other hand, there are 100 COVID-19 images and 3000 non-COVID-19 images in the test set. The distributions of the training and testing sets are presented in Table 2.

All chest radiographs are resized to 224×224 as the input to the deep neural networks. The last layer of each model is fine-tuned and trained for 100 epochs, and the batch size is 16. The learning rate is set to 0.0001, and the Adaptive Moment Estimation (Adam) optimizer was adopted to optimize the model training. The cross-entropy loss function was adopted for the training, which is defined as:

(1) $$L_{CE}=-\sum_{i=1}^{N}p_{i}\log q_{i}$$

where $p_{i}$ and $q_{i}$ denote the true and predicted probabilities for each sample, respectively. The hyperparameter settings of the deep neural networks are presented in Table 3.

**Figure 3:**
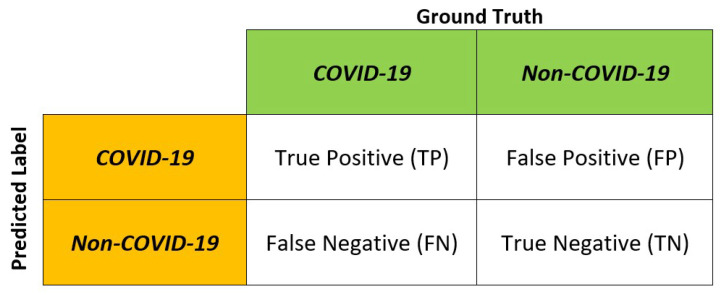
True Positive (TP), True Negative (TN), False Positive (FP) and False Negative (FN).

Several metrics are used in the performance evaluation of the deep neural networks—namely, the accuracy, precision, sensitivity (also known as recall), specificity and F1-score. The evaluation metrics are based on the True Positive (TP), True Negative (TN), False Positive (FP) and False Negative (FN) as depicted in Figure 3. The evaluation metrics are defined as:

(2) $$\begin{array}{ccc} \mathrm{Accuracy} & = & \frac{\mathrm{TP}\;+\;\mathrm{TN}}{\mathrm{TP}\;+\;\mathrm{FP}\;+\;\mathrm{TN}\;+\;\mathrm{FN}} \end{array}$$

(3) $$\begin{array}{ccc} \mathrm{Precision} & = & \frac{\mathrm{TP}}{\mathrm{TP}\;+\;\mathrm{FP}} \end{array}$$

(4) $$\begin{array}{ccc} \mathrm{Sensitivity} & = & \frac{\mathrm{TP}}{\mathrm{TP}\;+\;\mathrm{FN}} \end{array}$$

(5) $$\begin{array}{ccc} \mathrm{Specificity} & = & \frac{\mathrm{TN}}{\mathrm{FP}\;+\;\mathrm{TN}} \end{array}$$

(6) $$\begin{array}{ccc} F1-\mathrm{score} & = & \frac{2\times \mathrm{Precision}\times \mathrm{Sensitivity}}{\mathrm{Precision}+\mathrm{Sensitivity}} \end{array}$$

In this work, the Python programming language is engaged for the development of deep neural networks. Python contains many frameworks, libraries and extensions that facilitate the implementation of deep-learning models. The main libraries used are Tensorflow, Keras and Scikit-Learn.

TensorFlow is an open-source deep learning library developed by Google that is widely adopted in classification and regression tasks. Tensorflow is built in a modular manner, making it flexible and easily scalable. Another important library is Keras, where it provides the fundamental abstractions and building elements for designing and constructing the machine learning systems. As for the performance evaluation, various metric modules provided by Scikit-Learn, such as the accuracy, precision, recall, F1-score and confusion matrix are leveraged. The experiments were executed on Google Colaboratory with GPU enabled.

Table 4 demonstrates that all pre-trained deep neural networks achieved promising results. DenseNet201 yielded the highest accuracy where the model correctly labeled 98.58% of the COVID-19 and non-COVID-19 images. MobileNetV2 recorded the highest precision where 100% of the predicted COVID-19 images were correctly labeled. DenseNet201 achieved the highest sensitivity rate of 0.58. The sensitivity indicates the ability of the model in identifying the actual COVID-19 chest radiographs and is important in COVID-19 screening. MobileNetV2 achieved the highest specificity where it correctly labeled all non-COVID-19 test images.

**Figure 4:**
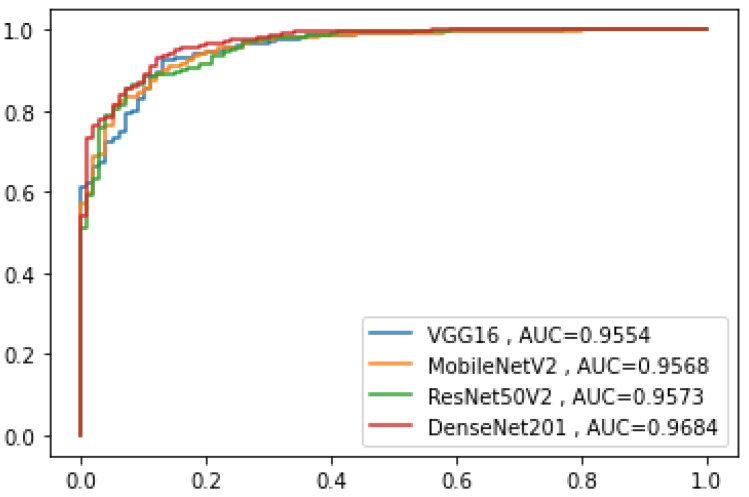
The receiver operating characteristics curve of the pre-trained models.

**Figure 5:**
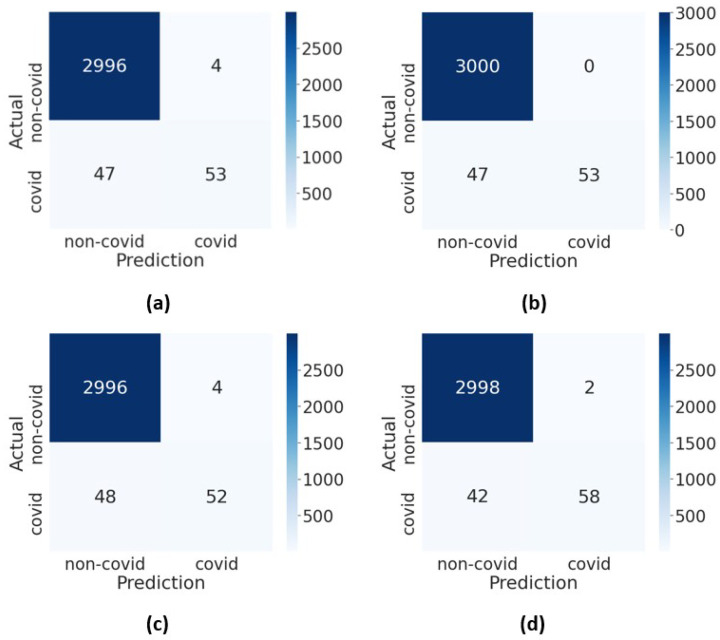
The confusion matrices of the pre-trained models: (**a**) VGG16, (**b**) MobileNetV2, (**c**) ResNet50V2 and (**d**) DenseNet201.

DenseNet201 recorded the highest F1-score at 0.725 with a good balance between precision and sensitivity. The training and testing times of the pre-trained models are also presented in the table. Figure 4 shows the receiver operating characteristics curve of the pre-trained models. The DenseNet201 has the highest area under the curve, which indicates that the model had relatively higher discriminating capability among the models. The confusion matrices of the pre-trained deep neural networks are presented in Figure 5.

Among the models in comparison, DenseNet201 generally outshined other models in comparison. Nevertheless, the models recorded sensitivity rates that are considered low in COVID-19 detection tasks. In view of this, some enhancements are proposed with the aim to improve the performance of the DenseNet201 model for COVID-19 diagnosis.

### 3.1. Data Augmentation

As COVID-19 is a relatively new disease, the current COVID-19 dataset suffers from image scarcity and class imbalance problems. In this work, data augmentation is leveraged to increase the number of training images by producing transformed variations of the images. Two data augmentation techniques—namely, random cropping and intensity normalization—were implemented to increase the size of the training set.

**Figure 6:**
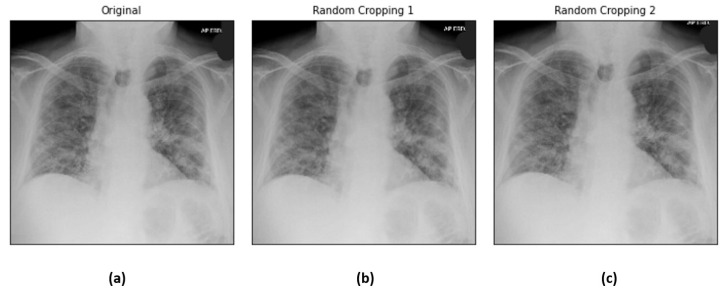
Sample chest X-ray images: (**a**) original, (**b**) random cropping (r−5,c−5) and (**c**) random cropping (r−10,c−10).

Random cropping produces synthetic images of smaller sizes (r−5,c−5) and (r−10,c−10), where (r,c) is the original image size. The small distortions produce more COVID-19 samples while preserving the prominent region of the chest radiographs. The output of random cropping is homogeneous to images with different distances between the focal spot and patient surface in radiography examinations. Figure 6 illustrates the sample chest X-ray images before and after random cropping.

Intensity normalization adjusts the intensity values of the original image I(x,y) into $I^{\prime }\left(x,y\right)$, as below:

(7) $$I^{\prime }\left(x,y\right)=\frac{I(x,y)-mean}{\sqrt{variance}}$$

**Figure 7:**
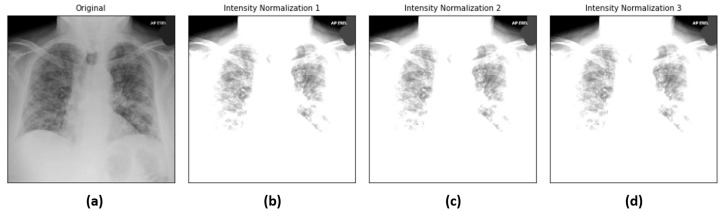
Sample chest X-ray images: (**a**) original, (**b**) intensity normalization (mean=0.485 and variance=0.229), (**c**) intensity normalization (mean=0.456 and variance=0.224) and (**d**) intensity normalization (mean=0.406,variance=0.225).

Three pairs of mean and variance values are used: (mean=0.485,variance=0.229), (mean=0.456 and variance=0.224) and (mean=0.406 and variance=0.225) [1]. The output of the intensity normalization is analogous to images with slight illumination variations. Figure 7 depicts the sample chest X-ray images before and after intensity normalization.

The data augmentation procedures increase the training set by a factor of 5, which means the number of COVID-19 chest radiographs increased from 84 to 420.

### 3.2. Class Weights

The dataset of COVID-Xray-5k dataset is highly imbalanced as there are 184 COVID-19 images and 5000 non-COVID-19 images. In order to solve class imbalance problems, several works [23,24] proposed sampling methods, either undersampling or oversampling. Undersampling reduces the samples from the majority class. In contrast, oversampling attempts to generate new samples for the minority class. However, sampling methods have their own drawbacks. Undersampling may cause the loss of representative samples, while oversampling may cause overfitting. In view of this, class weight is proposed to mitigate the class imbalance problem. The class weights are adjusted by placing more emphasis on the minority class, enabling the classifier to learn equally from all classes. The class weight is calculated as:

(8) $$\begin{array}{c} w_{j}=\frac{n_{samples}}{n_{class}\times n_{samples_{j}}} \end{array}$$

where

- $w_{j}$ denotes the weight for class *j*,
- $n_{samples}$ is the total number of samples,
- $n_{class}$ represents the number of class and
- $n_{samples_{j}}$ denotes the number of samples in class *j*.

In doing so, the weights are adjusted to be inversely proportional to the data size of each class, thus, mitigating the class imbalance problem.

### 3.3. Early Stopping

Another challenge of deep neural networks lies in the choice of the number of training epochs. The number of epochs affects the generalization capability and the performance of the deep neural networks. Too many epochs may result in the model overfitting the training dataset. On the other hand, too few epochs may lead to an underfitted model.

Both overfitting and underfitting impact the generalization capability and decay the performance of the model. In view of this, an early stopping strategy is adopted to halt the training once the model stops improving on the validation accuracy for a certain number of epochs. Subsequently, the model weights from the epoch with the highest validation accuracy will be restored.

### 3.4. Fine-Tuning

In transfer learning, the pre-trained model weights are redeployed to another smaller dataset by some modifications. In this work, the last five layers of DenseNet201 are unfrozen, and a new final classification layer is appended. These layers are subsequently trained on the COVID-Xray-5k dataset to fine-tune the higher-order feature representations, thus, tailoring the representations for COVID-19 detection.

**Figure 8:**
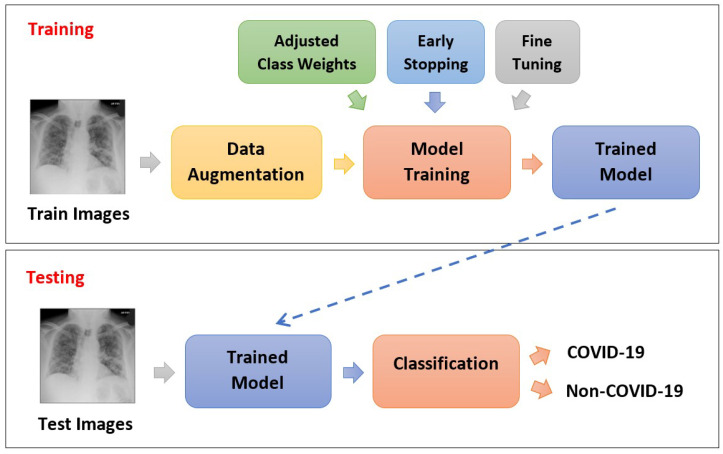
The process flow of the proposed COVID-19 diagnosis with enhanced DenseNet201.

With these enhancements, the overall process flow of model training and testing is illustrated in Figure 8.

## 4. Experimental Results

**Figure 9:**
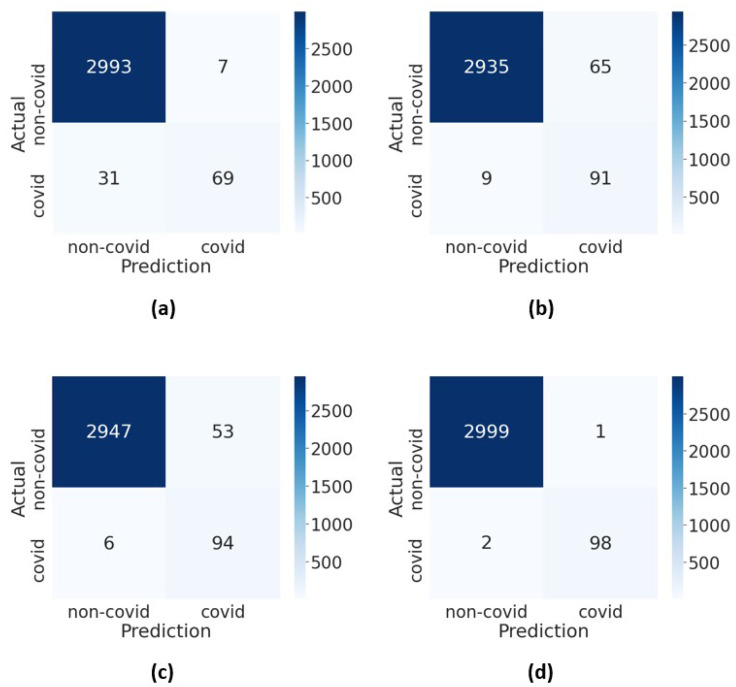
The confusion matrices of DensetNet201 with enhancements: (**a**) DenseNet201 with data augmentation, (**b**) DenseNet201 with data augmentation and adjusted class weights, (**c**) DenseNet201 with data augmentation, adjusted class weights and early stopping and (**d**) DenseNet201 with data augmentation, adjusted class weights, early stopping and fine-tuning.

The performance of DenseNet201 with enhancements was evaluated on the COVID-Xray-5k dataset. The last five layers of DenseNet201 were fine-tuned for 100 epochs, and the batch size was set to 16. In the training process, the data augmentation was deployed on the training set to increase the data size by a factor of 5. The training process was terminated once the validation accuracy stops improving for 15 epochs, and the weights with the highest validation accuracy were used. Not only that, the class weights were adjusted to be inversely proportional to the sample size of the COVID-19 and non-COVID-19 classes to compensate for the imbalanced class distribution. The confusion matrices of DenseNet201 with enhancements are presented in Figure 9.

As observed in Table 5, data augmentation slightly improved the performance of DenseNet201 in terms of the accuracy, sensitivity and F1-score. This is attributable to better discriminating power when more COVID-19 samples are generated for training. Although the accuracy slightly dropped when the adjusted class weights were added, the sensitivity improved tremendously. It is worth mentioning that the model sensitivity is crucial to ensure the early diagnosis of COVID-19 where the model is able to correctly identify COVID-19 samples.

The sensitivity of the model greatly increased from 0.69 to 0.91 when the class weights were adjusted to give more emphasis on the minority class. The improvement corroborates that adjusted class weights are effective in alleviating the bias caused by the skewed class distributions.

We also observed that incorporating early stopping avoids the overfitting problem and improves the overall performance of the model. To further improve the model for COVID-19 screening, fine-tuning the last few layers of DenseNet201 makes the learned feature maps more representative towards COVID-19 chest radiographs. The DenseNet201 model with data augmentation, adjusted class weights, early stopping and fine-tuning demonstrated outstanding classification accuracy, precision and F1-score despite the fact that limited COVID-19 samples were available.

The experimental results also exhibited high sensitivity and specificity rates, which are essential in COVID-19 screening. To further evaluate the performance of the enhanced DenseNet201 method, comparison with several state-of-the-art deep neural network methods was conducted as presented in Table 6.

Since the COVID-Xray-5k dataset is highly imbalanced, sensitivity and specificity are two important metrics for the comparison. Noticeably, the proposed method outperformed the state-of-the-art deep neural network methods. This is mainly due to the enhancements of data augmentation, class weights, early stopping and fine-tuning to the proposed method. Data augmentation increased the training samples and provided better representation to the model. In addition, the adjusted class weights help to mitigate the class imbalance problem. Furthermore, early stopping determined the best performing model and reduced the overfitting. Moreover, fine-tuning allowed the model to learn more robust representation towards the dataset.

## 5. Conclusions

Early diagnosis of the SARS-CoV-2 virus has become a challenge for scientists. The Polymerase Chain Reaction (PCR) test was introduced to detect the COVID-19 [25] patients. However, the PRC test is time-consuming, and it has an increased risk of false positives due to its lower specificity compared to culturing and staining [26]. In view of this, this paper evaluated four pre-trained deep neural networks—namely, VGG16, MobileNetV2, ResNet50 and DenseNet201—for COVID-19 diagnosis based on chest radiographs. The classification layer of the pre-trained models was replaced and trained on the COVID-Xray-5k dataset. DenseNet201 outshined the other models in comparison; however, the sensitivity rate was still moderate. To improve the robustness in early detection of COVID-19 cases, a high sensitivity rate is prominent. The sensitivity rate reflects how well the model can correctly recognize the COVID-19 samples. To this end, some enhancements on DenseNet201 are proposed.

The DenseNet201 model encourages feature reuse via dense connections among the layers within a dense block. The dense connections enhance the gradient flow and enable strong feature propagation through the layers. Furthermore, DenseNet201 uses much fewer trainable parameters, thus, improving the computational efficiency. In addition to that, some enhancements were incorporated into the DenseNet201 model. First, data augmentation was performed to address data scarcity problems due to the limited COVID-19 samples.

Secondly, the class weights were adjusted proportionally to alleviate the bias and skewness caused by the imbalanced class distribution. Thirdly, early stopping was leveraged to mitigate the overfitting problem while preserving the generalization capability of deep neural networks. Fourthly, fine-tuning was performed on the last few layers of DenseNet201 to better adapt the learned high-level features for the COVID-19 detection task. The empirical results demonstrate that DenseNet201 with these enhancements performed outstandingly despite the challenges posed by the dataset and deep neural network architecture.

Inspired by the promising performance of the attention-based models, such as Transformers, future works in COVID-19 diagnosis on chest X-rays could be done using attention-based models. As the regions in the chest X-rays that show the infections of COVID-19 are hardly perceivable, attention-based models are needed to assign higher significance to the infected regions, thus, improving the representation learning and classification of the model.

## Figures and Tables

**Table 1:** The summary of the existing works on COVID-19 diagnosis.

Reference	Method	Dataset	Accuracy (%)
Narin et al. (2020) [6]	ResNet50	341 COVID-19, 2800 normal	96.1
Basu et al. (2020) [7]	VGG16	305 COVID-19, 322 pneumonia, 350 normal, 50 others	90.13
Jain et al. (2021) [8]	Xception	576 COVID-19, 1583 normal and 4273 pneumonia	97 (F1-score)
Ismael et al. (2021) [9]	ResNet50, SVM	180 COVID-19, 200 normal	94.7
Wang et al. (2020) [10]	COVID-Net with PEPX	358 COVID-19, 13617 others	93.3
Sitaula and Hossain (2020) [11]	VGG16 with attention module	2138 images	87.49
Ahsan et al. (2021) [12]	VGG16	1979 COVID-19, 3111 normal	99.49
Abbas et al. (2021) [13]	DeTraC model	105 COVID-19, 80 normal, 15 SARS	93.1
Chakraborty et al. (2021) [14]	Corona-Nidaan	245 COVID-19, 5551 pneumonia, 8066 normal	95
Sharifrazi et al. (2021) [15]	Sobel filter, CNN, SVM	77 COVID-19, 256 normal	99.02
Aslan et al. (2022) [16]	Densenet201, SVM	219 COVID-19, 1341 normal, 1345 viral pneumonia	96.29
Tangudu et al. (2022) [17]	MobileNet with RSC	184 COVID-19, 5000 normal	99.71
Rehman et al. (2022) [18]	BA-ELM	184 COVID-19, 3000 normal	95.7

**Table 2:** The number of training and testing samples in the COVID-Xray-5k dataset.

Dataset	COVID-19	Non-COVID-19	Total Samples
Train Set	84	2000	2084
Test Set	100	3000	3100

**Table 3:** The hyperparameter settings of the deep neural networks.

Parameter	Values
Image size	224 × 224
Batch size	16
Dropout rate	0.2
Training epoch	50
Optimizer	Adam
Learning rate	0.0001

**Table 4:** The performance of the pre-trained deep neural networks on the COVID-Xray-5k dataset.

Deep Neural Network	Accuracy	Precision	Sensitivity	Specificity	F1-Score	Training Time (s)	Testing Time (s)
VGG16	0.9835	0.9298	0.53	0.9987	0.6752	921.21	17.07
MobileNetV2	0.9848	**1.0000**	0.53	**1.0000**	0.6928	464.10	6.29
ResNet50V2	0.9832	0.9286	0.52	0.9987	0.6667	503.23	13.03
DenseNet201	**0.9858**	0.9667	**0.58**	0.9993	**0.7255**	935.72	23.09

**Table 5:** The performance of DenseNet201 with different enhancements (DA = data augmentation, CW = adjusted class weights, ES = early stopping and FT = finetuning).

Enhancement	Accuracy	Precision	Sensitivity	Specificity	F1-Score
DenseNet201	0.9858	0.9667	0.58	0.9993	0.7250
DenseNet201 + DA	0.9877	0.9079	0.69	0.9977	0.7841
DenseNet201 + DA + CW	0.9761	0.5833	0.91	0.9783	0.7109
DenseNet201 + DA + CW + ES	0.9810	0.6395	0.94	0.9823	0.7611
DenseNet201 + DA + CW + ES + FT	0.9990	0.9899	0.98	0.9997	0.9849

**Table 6:** Comparison with the state-of-the-art deep neural network methods.

Method	Accuracy	Sensitivity	Specificity
GoogleNet [17]	0.9971	0.9971	0.9994
InceptionResNet [17]	0.9917	0.9917	0.9984
ResNet50 [17]	0.9986	**0.9986**	0.9988
MobileNet [17]	0.9932	0.9932	0.9996
MNRSC [17]	0.9971	0.9971	0.9988
BA-ELM [18]	0.9570	0.9870	0.7150
**Enhanced DenseNet201 (proposed)**	**0.9990**	0.9900	**0.9997**

## Data Availability

Not applicable.

## Abbreviations

The following abbreviations are used in this manuscript:

Adam	Adaptive Moment Estimation
TP	True Positive
TN	True Negative
FP	False Positive
FN	False Negative
